# Preoperative Hepatic Augmentation Versus Transarterial Chemoembolization for Hepatocellular Carcinoma With Insufficient Remnant Liver Volume: A Systematic Review and Meta‐Analysis

**DOI:** 10.1002/cam4.71050

**Published:** 2025-07-11

**Authors:** Wenjie Li, Hang Li, Qingyan Kong, Fei Teng, Zheyu Chen

**Affiliations:** ^1^ Department of Liver Surgery West China Hospital of Sichuan University Chengdu China

**Keywords:** hepatocellular carcinoma, meta‐analysis, preoperative hepatic augmentation, transarterial chemoembolization

## Abstract

**Background:**

Increasing remnant liver volume before major liver resection is an effective measure to reduce postoperative adverse events of hepatocellular carcinoma (HCC). We aimed to provide evidence for optimal management of HCC patients with insufficient future remnant liver volume (FRLV).

**Methods:**

A comprehensive search of various large medical databases, research registry platforms, and gray literature was performed up to May 2023. All comparative studies grouped by preoperative hepatic augmentation (PHA) and transarterial chemoembolization (TACE) were included. A random‐effects model was used for meta‐analysis, and the heterogeneity of the results was quantitatively assessed by funnel plots, sensitivity analyses, and subgroup analyses.

**Results:**

A total of eight comparative studies were selected for inclusion in this analysis, including 3523 patients. 5‐year overall survival (hazard ratio [HR] = 1.52, 95% confidence intervals [CI] = 1.07–2.15) and disease‐free survival (HR = 1.72, 95% CI = 1.40–2.10) were significantly different between the PHA and TACE groups. There was no significant difference between PHA and TACE with respect to 90‐day mortality, postoperative complication rate, or serious complication rate (*p* > 0.05). In subgroup analysis, compared with portal vein embolization, associating liver partition and portal vein ligation was highly associated with longer survival and fewer recurrences (*p* < 0.05). None of the above results exhibited obvious bias or heterogeneity.

**Conclusions:**

This study demonstrates that PHA allows for radical liver resection for HCC patients with insufficient FRLV without increasing the incidence of postoperative adverse events, which can effectively improve patient outcomes and delay tumor recurrence.

**Abbreviations**
ALPPS: associating liver partition and portal vein ligation for staged hepatectomy
CI: confidence intervals
DFS: disease‐free survival
FRLV/FRL: future remnant liver volume/future remnant liver
HCC: hepatocellular carcinoma
HR: hazard ratio
OS: overall survival
PHA: preoperative hepatic augmentation
PHLF: posthepatectomy liver failure
PRISMA: preferred reporting Items for systematic reviews and meta‐analyses
PSM: propensity score matching
PVE: portal vein embolization
RR: risk ratio
TACE: transarterial chemoembolization

## 1 Introduction

Primary liver cancer has become the sixth most diagnosed cancer and the third leading cause of cancer death worldwide. Hepatocellular carcinoma (HCC) is one of the most common types of primary liver cancer (approximately 75%–85% of cases) and is highly associated with chronic hepatitis virus infection (approximately 80%) [1, 2]. Liver transplantation has become the optimal option for the vast majority of HCC patients, but because of the shortage of liver donors and difficult matching, surgical resection beyond liver transplantation is the first‐line treatment option for preserving liver function and removing tumors in various large medical centers [3, 4]. However, HCC patients suitable for radical hepatectomy are not the majority, and the primary reason limiting the resection rate is posthepatectomy liver failure (PHLF) due to insufficient function of the future remnant liver (FRL) [5, 6]. There is a relationship between future remnant liver volume (FRLV) and remnant liver function, the former being easier to observe. Therefore, FRLV has become an important indicator for predicting postoperative liver function and assessing the risk of surgery [7, 8]. Currently, it is considered safe to perform hepatectomy with a FRLV/total standard liver volume of ≥ 20%, steatosis and cholestasis ≥ 30%–40%, and cirrhosis ≥ 50% in healthy livers [9].

For HCC patients with insufficient FLR, transarterial chemoembolization (TACE) or TACE combined with targeted pharmacologic immunotherapy is the guideline‐recommended local therapy and is also used as another pre‐ or posttreatment debulking or bridging therapy [10]. The rich blood supply of the hepatic artery in HCC and the blockage of the hepatic artery to induce tumor ischemia and necrosis are the therapeutic logic of TACE, but its treatment cycle is long and repetitive. Furthermore, TACE carries the risk of tumor progression or recurrence and severe liver damage. Thus, there is an ongoing search for other clinical regimens [11]. Preoperative hepatic augmentation (PHA) has been a promising discovery in recent years and stimulates compensatory hypertrophy in the FLR by selectively blocking the veins of the liver on the side of the tumor and enhancing the venous input to the contralateral liver, thereby expanding the indication range of hepatectomy for FRLV and reducing the risk of PHLF. Several operations have been developed or derived, among which portal vein embolization (PVE) and associating liver partition and portal vein ligation for staged hepatectomy (ALPPS) are currently the most commonly used clinical techniques. However, similar to TACE, PHA carries the risk of surgery‐related tumor progression and severe liver damage [12].

Currently, there are few relevant comparative studies comparing the efficacy and prognosis of two surgical strategies, TACE and PHA, in HCC patients. There is also a lack of relevant prospective studies, systematic reviews, and meta‐analyses, and conclusions differ among the few available studies. Thus, little is known about the optimal management of HCC patients with insufficient FRL. The aim of this study was to conduct a comprehensive systematic review of these two treatment strategies, compare the short‐ and long‐term prognosis of the two strategies, and conduct a meta‐analysis to provide evidence and recommendations for clinical treatment selection.

## 2 Methods

### 2.1 Specifications

This study was conducted in strict accordance with the requirements of the latest version of the guidelines for the Preferred Reporting Items for Systematic Reviews and Meta‐Analyses (PRISMA) and has been registered with the International Prospective Register of Systematic Reviews (http://www.crd.york.ac.uk/PROSPERO) [13]. Registration was completed under number CRD42023483984. No ethical issues and informed consent were involved in this study, and institutional ethics committee approval was not needed.

### 2.2 Search Strategy

In this study, a comprehensive search of the four major medical databases, PubMed, Embase, Cochrane Library, and Web of Science, was performed to identify all HCC studies published up to May 2023 that compared the TACE and PHA. No additional restrictions on study type, language, or country were imposed on the literature search. In the PubMed database, the specific formula was ((((((hepatocellular[Title/Abstract]) OR (liver[Title/Abstract])) AND (((carcinoma*[Title/Abstract]) OR (cancer*[Title/Abstract])) OR (metasta*[Title/Abstract]))) OR (hepatoma*[Title/Abstract])) OR (“Carcinoma, Hepatocellular”[Mesh])) AND ((((“associating liver partition and portal vein ligation for staged hepatectomy”[Title/Abstract]) OR (ALPPS[Title/Abstract])) OR (portal vein embolization[Title/Abstract])) OR (PVE[Title/Abstract]))) AND ((transcatheter arterial chemoembolization[Title/Abstract]) OR (TACE[Title/Abstract])). Other databases were searched using the same subject headings (MeSH) and keywords. Relevant studies within the clinical trials.gov and NEAR websites were also manually entered after manual screening. In addition, for relevant reviews and studies considered for inclusion, the reference lists were manually crosssearched, and eligible studies were manually screened. Although series from the same author or center were screened, only the publication with the most complete data was included.

### 2.3 Inclusion and Exclusion Strategies and Criteria

Screening of all retrieved results was performed independently by two independent investigators (Wenjie Li and Hang Li) using predetermined inclusion and exclusion criteria. The initial screening was completed by reading the titles and abstracts, followed by a secondary screening involving full‐text reading, which then determined the final included studies. In the case of disagreement during the criteria development and screening, the decision for inclusion or exclusion was made after discussion with a third investigator (Qingyan Kong).

Inclusion criteria: (1) Adult study subjects (aged ≥ 18 years); (2) Confirmed HCC and insufficient FRLV assessed preoperatively; (3) A prospective or retrospective study of TACE and PHA (ALPPS/PVE); (4) Comparative studies, that is, non‐single arm studies; and (5) At least several of the following outcomes have been reported: incidence or number of postoperative complications (including PHLF), 90‐day mortality, and overall survival (OS).

Exclusion criteria: (1) Reviews, letters, editorials, case reports, or abstracts; (2) Nonhuman studies; (3) Studies involving patients with non‐HCC disease; and (4) Studies without full text or data of interest.

### 2.4 Outcomes and Extraction of Interest

Wenjie Li and Hang Li independently captured data from the main text, figures, and attachments of the included literature in six dimensions: (1) study label: title, author, year, country, study population, type, grouping, etc.; (2) patient baseline data: sex, age, number, and diameter of tumors, metastasis, test results (e.g., AFP, ICG‐R15), classification (e.g., BCLC classification, Child–Pugh score), etc.; (3) pre‐ and postliver volume changes: FRLV, FRLV/SLV, kinetic growth rate, etc.; (4) operative data: operative time, operative interval, positive resection margin rate, etc.; (5) short‐term outcomes: 90‐day mortality, postoperative complications and overall incidence; (6) long‐term outcomes: OS and disease‐free survival (DFS), hazard ratio (HR), etc. Of the data collected, the long‐term outcome (6) was the primary outcome of interest for this study, and the remainder were included as secondary outcomes. For the few long‐term prognostic indicators that were not directly reported, in addition to sending mail to the study authors or institutions requesting their raw data, we also referred to the study by Tierney, JF, and Wenjie Li, Hang Li, and Qingyan Kong used the Engauge Digitizer software (version 11.1) according to the Kaplan–Meier curve enlargement plots provided by the literature for relevant estimation points and calculations [14].

### 2.5 Literature Quality Evaluation

The quality of the included studies was evaluated using the modified Newcastle–Ottawa scale. Depending on the type of studies, the reviews were star scored from three angles (Selection, Comparability, and Outcome) with eight questions and up to nine stars. A score ≥ seven stars was considered a high‐quality study, and the process was independently completed by Wenjie Li and Hang Li [15, 16].

### 2.6 Statistical Analysis

Meta‐analysis was performed using StataCorp LLC software (version 17.0, USA). The risk ratio (RR, dichotomous variable), HR (dichotomous variable) and their 95% confidence intervals (CI) were reported for each study, and *p* < 0.05 was considered statistically significant. All statistical analyses were performed using a random effects model, and *I* ^2^ was used to measure study heterogeneity when evaluating RR or HR, with *I* ^2^ over 50% considered significant heterogeneity among studies (*p* > 0.05), < 30% considered no heterogeneity, and 30%–50% considered moderate heterogeneity [17]. Where significant heterogeneity was present, a sensitivity analysis was employed for each study to observe the effect of individual studies on the outcome, screening for sources of heterogeneity. This study analyzed the potential publication bias among the results by funnel plot and Galbraith plot observation, and the symmetry of the funnel plot was quantitatively evaluated with the results of Egger's test. In addition, subgroup analysis was performed according to the type of PHA surgery (PVE and ALPPS) to investigate the differences between different PHA types for TACE.

## 3 Results

### 3.1 Search Results and Description

**FIGURE 1:**
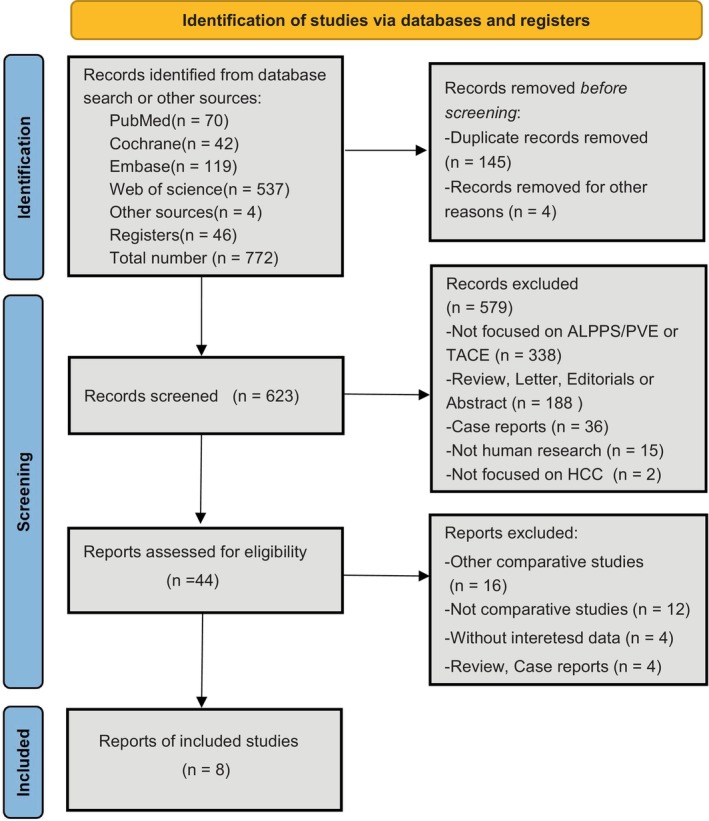
The flow diagram for search results and study screening (PRISMA 2020 flow diagram for new systematic reviews).

In accordance with the requirements of the 2020 new version of PRISMA, a total of 772 articles were retrieved from the aforementioned databases, research registry platforms, and websites. 149 studies were removed due to duplication or series, and after two screenings, only eight studies fully met the inclusion and exclusion criteria (Figure 1). The eight included studies [18, 19, 20, 21, 22, 23, 24, 25] collected a total of 3523 HCC patients, and the majority used propensity score matching (PSM). Thus, the final propensity was to compare 531 patients: 256 (48.2%) patients treated with PHA and 275 (51.8%) patients treated with TACE. Because the included studies were all retrospective cohort studies, the questions and criteria for assessing cohort studies in the modified Newcastle–Ottawa scale were adopted. Seven studies had NOS scores ≥ seven stars; therefore, all but one study was considered high quality (Table 1).

**TABLE 1:** Methodological quality of included retrospective cohort studies.^a^

Study (year)	Selection				Comparability of cohorts	Outcome			Quality score
	Exposed cohort	Non exposed cohort	Ascertainment of exposure	Outcome of interest		Assessment of outcome	Follow‐up long enough	Adequacy of follow up	
Haoqi et al. (2022)	★	★	★	★	★★	★	★	☆	★★★★★★★★☆
Zheng et al. (2020)	★	★	★	★	★★	★	★	★	★★★★★★★★★
Zhenfeng et al. (2021)	★	★	★	★	★★	★	☆	★	★★★★★★★★☆
JiaHui et al. (2022)	★	★	★	★	★★	★	★	☆	★★★★★★★★☆
Lixin et al. (2020)	★	★	☆	★	★★	★	★	★	★★★★★★★★☆
Chihan et al. (2019)	★	★	★	★	★★	★	☆	☆	★★★★★★★☆☆
Dong et al. (2007)	☆	★	☆	★	★☆	★	★	★	★★★★★★☆☆☆
Gil et al. (2020)	★	★	★	★	★☆	★	★	☆	★★★★★★★☆☆

^a^ Assessed by the modified Newcastle‐Ottawa scale, criteria for award of stars described in the panel.

### 3.2 Patient Baseline

The eight included studies were all comparative studies, and effective baseline comparability was observed. Six studies [18, 19, 20, 21, 22, 23] adopted PSM's method to quantitatively evaluate the variable balance between matched groups to exclude patient selection bias. The *p* values of each baseline data point were > 0.05 between matched groups, and the differences were statistically insignificant and comparable. Dong et al. [24] applied Fisher's exact test, paired *t* test, and others to contrast each baseline between the two groups, with the exception of age (*p* = 0.04 < 0.05), which had a baseline *p* value of more than 0.25. Gil Chun Park et al. [25] confirmed baseline comparability between the two groups one by one by the same method (*p* > 0.05). In summary, all studies included in this meta‐analysis exhibited comparable baseline data, and the specific data and reporting forms are presented in Table 2.

**TABLE 2:** Characteristics of the studies included.

Study	Country	Year/Study period	Design/Centers	Cancer diagnosis	Intervention/Control	Analysis	Patients: AP/TA	Sex (M‐F): AP/TA	Age: AP/TA	No of tumors (S‐M): AP/TA	Diameter (cm): AP/TA	Vascular invasion: AP/TA	Distant metastasis: AP/TA	Lymphatic node metastasis: AP/TA
Haoqi et al.	China	2022/2014.8–2021.1	R/Single	Hepatocellular carcinoma	ALPPS/TACE	PSM	24/330	24–0/24–0	49.55 ± 11.75/49.05 ± 17.26	14–8/14–8	7.37 ± 3.66/8.53 ± 4.92	6/5	None/None	None/None
Zheng et al.	China	2020/2013.4–2017.9	R/Single	Hepatocellular carcinoma	ALPPS/TACE	PSM	45/1305	40–5/39–6	52 (24–67)/55 (33–81)	28–17/28–17	12.5 (6–31)/12.0 (1–22)	17/20	None/None	None/None
Zhenfeng et al.	China	2021/2017.1–2019.12	R/Single	Hepatocellular carcinoma	ALPPS/TACE	PSM	20/66	17–3/19–1	47 (32–75)/49 (26–73)	20–0/20–0	14.5 (10–20.5)/14.9 (10.2–25)	11/11	None/None	NR/NR
JiaHui et al.	China	2022/2014.8–2020.7	R/Three	Hepatocellular carcinoma	ALPPS/TACE	PSM	54/184	50–4/52–2	50 (23–72)/49 (22–73)	17–37/15–39	10.2 (3.9–22.2)/12.2 (3–22)	29/34	None/None	None/None
Lixin et al.	China	2020/2013.11–2018.6	R/Single	Hepatocellular carcinoma	ALPPS/TACE	PSM	23/76	22–1/NR	40 (32–66)/41 (26–71)	12–11/7–7	8.8 (2.3–16.6)/8.15 (5.5–16.8)	7/5	None/None	5/NR
Chihan et al.	China	2019/2014.8–2018.8	R/Single	Hepatocellular carcinoma	ALPPS/TACE	PSM	20/1105	19–1/19–1	48.6 ± 8.5/51.4 ± 15.89	15–5/14–6	10.1 ± 4.2/10.0 ± 3.9	NR/NR	NR/NR	NR/NR
Dong et al.	Korea	2007/1999.1–2002.12	R/Single	Hepatocellular carcinoma	PVE/TACE	Comparison	32/64	26–6/53–11	51.4 ± 10.6/57.8 ± 8.2	23–9/47–17	6.0 ± 2.8/5.8 ± 2.8	10/19	None/None	None/None
Gil et al.	Korea	2020/1993.11–2017.11	R/Single	Hepatocellular carcinoma	PVE/TACE	Comparison	38/28	34–4/24–4	54.71 ± 8.51/51.5 ± 9.03	35–3/24–4	5.94 ± 3.23/8.69 ± 3.58	5/11	NR/NR	NR/NR

AFP (ng/mL): AP/TA	TBIL (μmol/L): AP/TA	ALT (U/L): AP/TA	ALT (U/L): AP/TA	ICG‐R15 (%): AP/TA	MELD: AP/TA	BCLC classification: AP/TA	Child‐Pugh: AP/TA	METAVIR: AP/TA	Surgical success rate: AP/TA	Negative margin: AP/TA	Median follow‐up (month): AP/TA	Type of survival outcomes: AP/TA
4280 ± 10,211/3616 ± 9986	NR/NR	NR/NR	NR/NR	9.67 ± 2.74/NR	7.44 ± 1.46/6.75 ± 3.45	5‐10‐9/NR	NR/NR	NR/NR	91.7%/NR	NR/NR	NR/NR	1, 3, 5 year‐OS; 1, 2, 5 year‐DFS/1, 3, 5 year‐OS
402 (2–60,500)/494 (1–60,500)	NR/NR	NR/NR	NR/NR	NR/NR	3.9 (1–10.3)/3.39 (1–6.9)	19‐9‐17/NR	5 (5‐6)/5 (5‐6)	2‐10‐11‐5‐13‐4/NR	91.1%/NR	100.0%/NR	16.0 (0.1–46.9)/NR	1, 3 year‐OS+DFS/1, 3 year‐OS+DFS
12 (≥ 400)/12 (≥ 400)	NR/NR	NR/NR	NR/NR	4.4 (2.4–10.9)/NR	5 (2–11)/6 (2–10)	7‐5‐8/NR	19‐1‐0/19‐1‐0	2‐1‐2‐3‐12‐0/NR	100.0%/NR	100.0%/NR	21.3 (12.1–41.6)/NR	1, 3 year‐OS+PFS/1, 3 year‐OS+PFS
33 (≥ 400)/32 (≥ 400)	NR/NR	NR/NR	NR/NR	NR/NR	5.03 (0.08–11.56)/0 (≥ 12)	0‐25‐29/NR	52‐2‐0/51‐3‐0	0‐0‐0‐0‐45‐0/NR	83.3%/NR	100.0%/NR	15.8 (0.7–92.9)/6.05 (0.1–76.0)	1, 3, 5 year‐OS+DFS/1, 3, 5 year‐OS
2306 (45–139,031)/659 (5–118,900)	15.1 (8–23.6)/13.5 (4.1–42.3)	38.5 (32–59)/31.5 (19–157)	37 (17–320)/34.5 (10–218)	NR/NR	NR/NR	7‐6‐10/NR	NR/NR	0‐4‐5‐3‐7‐4/NR	87.0%/NR	NR/NR	NR/NR	1, 3, 5 year‐OS; 1, 2, 5 year‐DFS/1, 3, 5 year‐OS
NR/NR	35.2/20.1	65.1/48.6	71.7/66.3	NR/NR	5.88/6.03	11‐4‐5/11‐3‐6	19‐1‐0/18‐2‐0	1‐0‐5‐4‐10‐0/NR	100.0%/NR	NR/NR	NR/NR	1, 3 year‐OS/1, 3 year‐OS
1.59 ± 1.2/1.78 ± 1.3 (log10 ng/mL)	NR/NR	NR/NR	NR/NR	9.1 ± 6.2/NR	NR/NR	NR/NR	NR/NR	0‐0‐0‐12‐17‐3/NR	100.0%/NR	100.0%/NR	64 (12–94)/52 (6–96)	1, 3, 5 year‐OS+DFS/1, 3, 5 year‐OS+DFS
4.1 (2.7–16.5)/306.5 (16.8–9024)	0.7 (0.6–0.8)/0.7 (0.5–0.95) (mg/dL)	NR/NR	NR/NR	11.8 (6.8–15.2)/10.5 (7.3–14.5)	NR/NR	NR/NR	NR/NR	0‐0‐0‐0‐9‐29/0‐0‐0‐0‐11‐17	100.0%/NR	100.0%/NR	NR/NR	1, 3, 5 year‐OS+RFS/1, 3, 5 year‐OS+RFS

*Note:* Data shown represents mean ± standard deviation or median (minimum–maximum).

Abbreviations: ALT, alanine aminotransferase; AP, ALPPS & PVE group; AST, aspartate aminotransferase; BCLC, Barcelona Clinic Liver Cancer = (Early‐Intermediate‐Advanced); Child‐Pugh, (A‐B‐C); F, female; ICG‐R15, indocyanine green retention rate at 15 min; M, male; M, multiple tumors; MELD, The Model of End‐Stage Liver Disease score; METAVIR staging of liver fibrosis, (no fibrosis or cirrhosis‐mild fibrosis‐moderate fibrosis‐severe fibrosis‐cirrhosis‐unclear or no report); No, number; NR, not report; PSM, propensity score matching; R, retrospective; S, single tumor; TA, TACE group; TBIL, total bilirubin.

### 3.3 Primary and Secondary Outcomes

Raw data for all primary and secondary outcomes were collected by standard extraction (Tables S1–, S3, you can find all the complete data online) and meta‐analysis (Table 3). All studies reported full data in the PHA group, but some relevant data was missing in the TACE group, primarily in studies adopting PSM. Eight studies reported OS every year up to 5 years, and no significant difference was observed in OS at any year except 5 years (RR 1.64, 95% CI [1.04, 2.60], *p* = 0.034). However, merged OS differed significantly between the groups (RR 1.30, 95% CI [1.07, 1.59], *p* = 0.009), with an *I* ^2^ of 38.8%. Therefore, patients in the PHA group had a longer survival than those in the TACE group. Four studies [19, 20, 24, 25] reported DFS; with the exception of 5‐year DFS (RR 1.57, 95% CI [1.00, 2.46], *p* = 0.048), DFS did not differ between the two groups. However, merged DFS differed significantly (RR 1.52, 95% CI [1.24, 1.88], *p* = 0.000) with an *I* ^2^ of 0.0%. These results suggested that patients in the PHA group experienced fewer recurrences than those in the TACE group. For short‐term outcomes, for example, 90‐day mortality, postoperative complications, and severe complications, the RR values were not significantly different (*p* > 0.05) between the PHA and TACE groups, although they were all > 1.

**TABLE 3:** Results of meta‐analysis comparison.

Outcomes of interest	Studies	No of patients		RR (95% CI)	*Z*	*p*	Study heterogeneity					
		AP group	TA group				X^2^	df	*p*	*I* ^2^ (%)	*p* (%)	DL (Tau^2^)
Long‐term outcomes												
Overall survival	8	244	266	1.30 (1.07, 1.59)	2.603	0.009	6.53	4	0.163	38.8	78.7	0.0196
1‐year OS	8	244	266	1.08 (0.95, 1.24)	1.162	0.245	10.24	7	0.175	31.7	71.2	0.0110
2‐year OS	8	244	266	1.39 (0.98, 1.96)	1.869	0.062	22.70	7	0.002	62.9	88.5	0.1422
3‐year OS	8	244	266	1.66 (0.97, 2.86)	1.840	0.066	28.92	7	0.000	75.8	91.5	0.3750
4‐year OS	4	145	167	1.41 (0.85, 2.36)	1.322	0.186	7.40	3	0.060	59.5	88.6	0.1383
5‐year OS	4	145	167	1.64 (1.04, 2.60)	2.124	0.034	4.36	3	0.225	31.2	79.2	0.0669
Recurrence‐free survival	4	135	157	1.52 (1.24, 1.88)	3.967	0.000	1.00	4	0.910	0.0	2.0	0.0000
1‐year RFS	4	135	157	1.61 (0.96, 2.69)	1.803	0.071	10.33	3	0.016	71.0	91.6	0.1754
2‐year RFS	3	90	112	1.45 (0.99, 2.11)	1.926	0.054	2.08	2	0.353	4.1	74.0	0.0052
3‐year RFS	4	135	157	2.19 (0.94, 5.11)	1.818	0.069	8.78	3	0.032	65.8	90.7	0.3998
4‐year RFS	2	70	92	1.40 (0.93, 2.11)	1.624	0.104	0.16	1	0.693	0.0	0.0	0.0000
5‐year RFS	2	70	92	1.57 (1.00, 2.46)	1.975	0.048	0.01	1	0.903	0.0	0.0	0.0000
Short‐term outcomes												
90‐day mortality	3	84	112	3.53 (0.62, 20.01)	1.424	0.154	0.08	1	0.777	0.0	0.0	0.0000
Morbidity	4	125	254	1.07 (0.39, 2.94)	0.132	0.895	29.75	3	0.000	89.9	96.9	0.9411
Morbidity (≥Grade‐IIIb)	4	125	254	1.62 (0.50, 5.23)	0.809	0.418	5.32	3	0.150	43.7	82.7	0.5882

Abbreviations: AP, ALPPS & PVE group; CI, confidence interval; df, degrees of freedom; HR, hazard ratio; No, number; NR, not report; OR, odds ratio; RR, risk ratio; SMD, standard mean difference; TA, TACE group.

**FIGURE 2:**
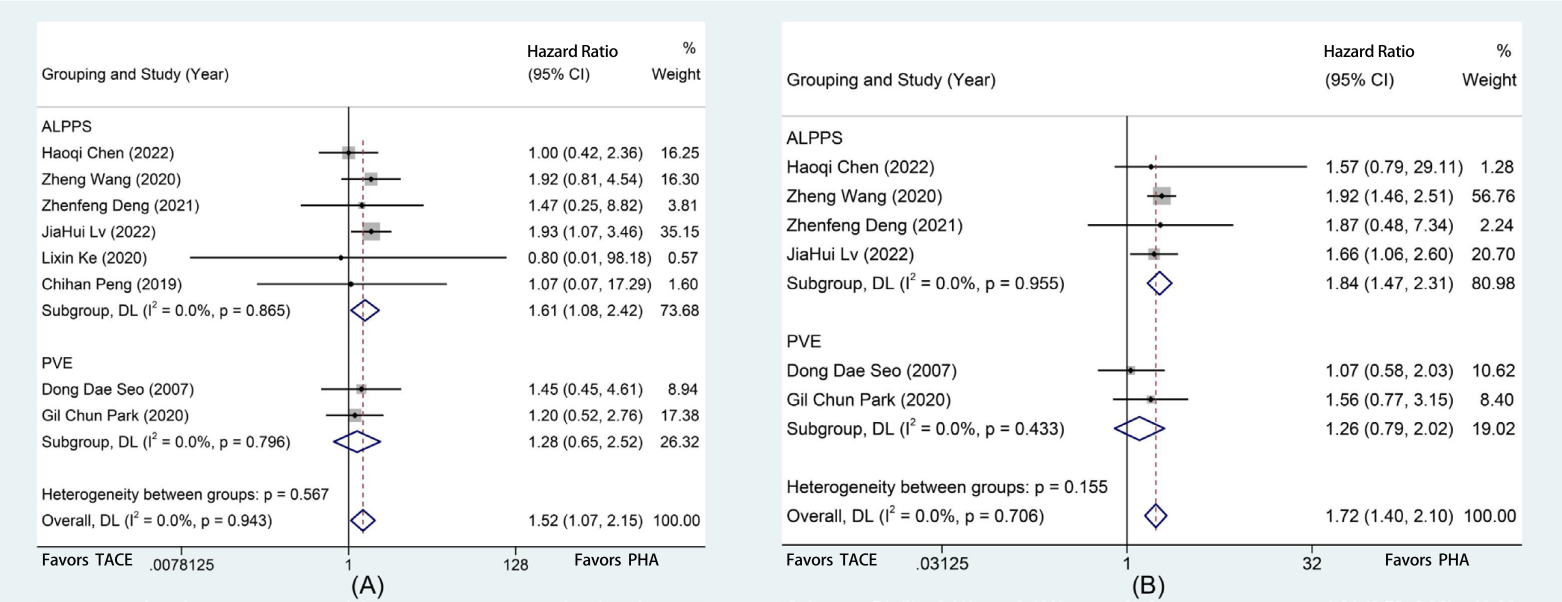
Outcomes of the subgroup Meta‐analysis (according to the different surgery types for PHA). (A) Meta‐analysis of HRs (OS). (B) Meta‐analysis of HRs (DFS).

Kaplan–Meier survival curves were combined for continuous survival analysis of OS and DFS for all studies, with effect size defined as HR. Forest plots for statistical analysis are presented in Figure 2. The HR (OS) was 1.52 (95% CI [1.07, 2.15], *p* = 0.019), and the HR (DFS) was 1.72 (95% CI [1.40, 2.10], *p* < 0.001); all differed significantly. The above results suggest that PHA is superior to TACE with respect to both long‐term survival and recurrence‐free survival for HCC patients with insufficient FRLV. There was no obvious difference between PHA and TACE in terms of adverse events such as 90‐day mortality, postoperative complications, or severe complication rates.

### 3.4 Subgroup Analysis

We performed a subgroup analysis for the two types of PHA currently most widely used clinically, ALPPS and PVE, to compare the differences between the two separately and to compare the differences with TACE (Figure 2). Analytical data demonstrated the significant superiority of ALPPS for long‐term survival (HR 1.61, 95% CI [1.08, 2.42], *p* = 0.021) and recurrence‐free survival (HR 1.84, 95% CI [1.47, 2.31], *p* < 0.001) compared with TACE. However, PVE did not significantly differ from TACE with respect to either long‐term survival (HR 1.28, 95% CI [0.65, 2.52], *p* = 0.476) or recurrence‐free survival (HR 1.26, 95% CI [0.79, 2.02], *p* = 0.327).

### 3.5 Publication Bias and Heterogeneity Analysis

**FIGURE 3:**
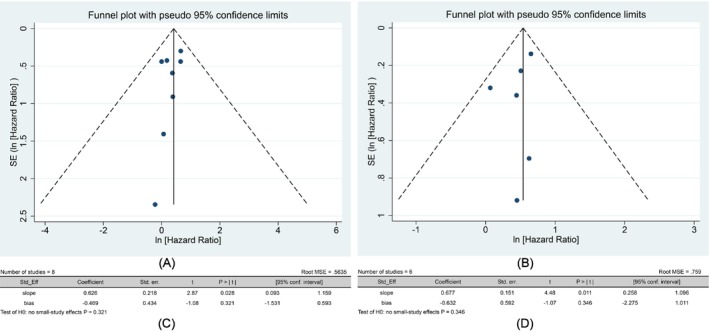
Results of the analysis for publication bias. (A) Funnel plot of HRs (OS). (B) Funnel plot of HRs (DFS). (C) Egger's test of HRs (OS). (D) Egger's test of HRs (DFS).

**FIGURE 4:**
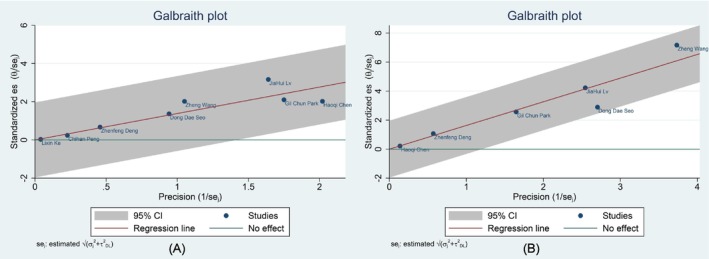
Heterogeneity measured by the Galbraith plots of HRs (OS) (A) and HRs (DFS) (B).

Publication bias and heterogeneity of the primary outcome and continuous survival analysis were analyzed by funnel plots, Egger's quantitative test, and Galbraith plots (Figures 3 and 4). HRs (OS and DFS) were used as the primary analysis effect sizes. All funnel plots were visually observed to be symmetrical, and no studies with obvious bias were observed. This was also confirmed by the results of Egger's test for quantitative analysis (all *p* > 0.05); there was no obvious publication bias for all results. The Galbraith plot intuitively exhibited the results of heterogeneity analysis. All results were located within the gray area; there was no obvious heterogeneity, and the slope between different loci was close to the red line passing through the origin, as was the homogeneity among studies.

### 3.6 Sensitivity Analyses

**FIGURE 5:**
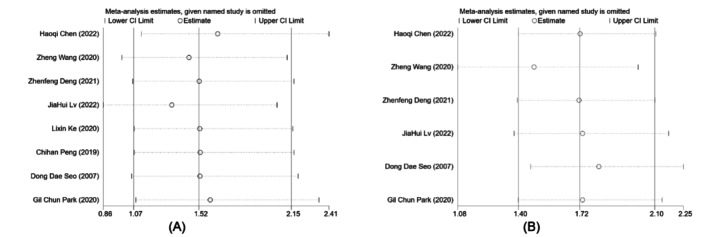
Sensitivity analysis performed by excluding single studies of HRs (OS) (A) and HRs (DFS) (B).

We conducted a sensitivity analysis for each study, including a change of effect model and exclusion of single studies to test the stability of the results of the primary outcome analysis. When changing the random effects model to a fixed effects model, the statistical significance of all results did not change. After excluding Jiahui et al. [21], the results of HRs (OS) were no longer statistically significant (HR 1.33, 95% CI [0.86, 2.05]), but this did not occur when the remaining single studies were removed. Thus, the validity of the conclusion remained, and none of the results of HRs (DFS) changed statistically significantly when any single study was removed (Figure 5). Therefore, the analysis results were stable to a certain degree.

## 4 Discussion

Most HCC treatments have a damaging effect on the liver and easily aggravate underlying liver disease [26]. Radical liver resection is the optimal solution for the vast majority of patients, but a condition prone to FRLV insufficiency occurs when the extent of resection is large. This in turn leads to a greatly increased risk of postoperative liver dysfunction and even PHLF in patients [27]. However, such patients often lack options in clinical decision‐making. TACE combined therapy is the current recommended regimen in addition to conservative treatment [28]. A recent series by Meta suggested that the long‐term prognosis of TACE was lower than expected compared with conventional hepatic resection, and the related regimens of TACE combined with hepatic resection similarly had no significant advantage in long‐term OS and DFS [29, 30, 31].

This appears to have improved in recent years due to the excellent performance of PHA in clinical practice, with PVE and ALPPS becoming progressively accepted by clinicians for inclusion in clinical decision‐making as optional treatment options for FRLV insufficiency. Essentially, PHA and TACE are “compromises” to the higher morbidity risk of PHLF, and hepatectomy remains one of the most effective radical measures for HCC patients. Only when the harms of hepatectomy outweigh the benefits when assessed preoperatively will PHA and TACE become attractive “alternatives.” However, for the same alternative treatment, studies comparing the prognostic difference between the PHA and TACE are scarce, and a summary of relevant experience is lacking. This article is the first systematic review and meta‐analysis to systematically compare the long‐term prognostic impact of PHA and TACE in FRLV‐insufficient HCC patients.

The liver has an astonishing regenerative capacity, and after hepatectomy, the increased release of multiple intrahepatic growth factors and hormones induced by injury stimuli promotes regeneration. In addition, multiple changes in the portal venous system have been considered enabling factors that activate liver regeneration [32, 33]. After resection, the liver volume decreases, and the arterial supply also decreases in an equal proportion, but the portal blood flow remains largely unchanged. Therefore, the portal blood flow received by the remnant liver suddenly increases. A relative decrease in arterial supply results in decreased oxygen delivery, whereas an increase in portal blood flow results in increased delivery of various intestinally derived growth factors (e.g., epidermal growth factor, tumor necrosis factor) or hormones (e.g., insulin) that stimulate liver regeneration [34, 35]. Taken together, portal vein alterations greatly accelerate liver regeneration after resection, which shares similarities with the typical wound healing process [36].

PHA cleverly takes advantage of the important influence of the portal vein during liver regeneration, mimics the blockade of the portal vein during hepatectomy, promotes hypertrophy of the FRL that is not wavered by tumor, and creates favorable conditions for second‐stage radical hepatectomy. PVE, currently the most widely used PHA method, selectively plugs the portal vein several weeks before major liver resection, artificially contributes to the blood supply to the remnant liver after similar resection, and stimulates liver regeneration. Numerous studies have demonstrated its safety and efficacy [37]. Currently, as a successful preoperative strategy, PVE is not without limitations: the surgical interval between PVE and second‐stage resection is long, there is a possibility of tumor progression during the period, and there is a lack of corresponding treatment measures. Moreover, some patients do not achieve the requirements for second‐stage resection in FRLV after PVE [38]. The prevailing view currently holds that a series of mechanisms that promote hepatocyte regeneration will similarly act on tumor cells through the collateral circulation or tissue microenvironment of the intact liver, which, on the one hand, promotes tumor progression. However, on the other hand, the liver to be resected shunts the growth‐promoting effect of the remnant liver and is an important cause of insufficient hypertrophy of the FRL [39, 40].

To solve these problems, on the basis of PVE, the FRL and the liver to be resected are completely or partially disconnected, blocking or reducing the possible pathway between the remnant liver and the tumor, and this “incomplete liver resection” is ALPPS [41]. ALPPS is undoubtedly successful in reducing the surgical interval, preventing tumor progression and promoting FRL hypertrophy, but the larger injury makes its postoperative morbidity and mortality significantly higher than PVE, which greatly limits the application of ALPPS [42, 43].

Both long‐term prognostic analysis of OS and DFS within 5 years and continuous survival analysis of HRs (OS) and HRs (DFS) revealed the obvious advantages of PHA compared with TACE. Furthermore, there were no significant differences between PHA and TACE in terms of adverse events such as 90‐day mortality, postoperative complications, or incidence of severe complications. Our results suggest that in HCC patients with insufficient FRLV for whom major liver resection is contraindicated, PHA can improve the surgical resectability rate, prolong patient survival time, and reduce the probability of postoperative recurrence without increasing the incidence of adverse events and represents a better treatment option than TACE.

In addition, because surgical approaches and operations for PHA differ and their advantages and disadvantages vary, we performed subgroup analyses separately for the long‐term prognosis of PVE and ALPPS. In comparison with TACE, ALPPS maintains significant advantages with respect to HRs (OS and DFS), but PVE exhibits no obvious difference compared with TACE. Our results suggest that ALPPS would be more suitable than PVE and TACE for HCC patients with insufficient FRLV, but the interpretation of this result requires caution. First, there were only two studies in the PVE group in the subgroup analysis, and the related data were lacking and prone to bias, making the conclusion inconsistent with reality. Second, we did not perform a corresponding subgroup analysis for adverse events such as mortality and postoperative complications, primarily due to missing data. However, combined with the reports of long‐standing PHA series, ALPPS tends to have a high complication rate and mortality. A 2018 RCT from a high‐volume center by Sandstrom P reported that although ALPPS confers a significant advantage in terms of surgical resection rates (92% vs. 57%), it is often associated with a higher 90‐day mortality [42]. A review by Lang Hauke similarly demonstrated that ALPPS, although improving resectability, has often been noted to exhibit a nonnegligible problem in terms of safety and oncological benefit, and improving ALPPS technology, minimizing the scope of one‐stage surgery, and improving safety remain the primary development goals [44]. Tustumi Francisco included 43 single‐arm studies and three comparative studies for meta‐analysis targeting different strategies of PHA, and the results suggested that ALPPS was associated with a high risk of PHLF, major complications, and mortality [45]. Thus, in our analysis, ALPPS exhibited excellent performance with respect to long‐term prognosis most likely at the cost of a higher risk of adverse events, which would reduce the therapeutic utility of ALPPS.

The present study has several interesting characteristics. First, at present, PHA is primarily applied in metastatic liver cancer, especially in the case of colorectal cancer liver metastases, whereas there are few studies of PHA in HCC patients. We sought to make a first collation and induction. Second, all studies included in this review exhibited comparable baseline patient characteristics. Six studies used the paired PSM, and the remaining two quantitatively assessed the baseline data and reported the corresponding *p* values. Third, the results of each analysis were also examined qualitatively and quantitatively for publication bias, heterogeneity, and sensitivity, and the vast majority of the results did not exhibit publication bias or heterogeneity.

Despite the analysis of valuable results, there remain some weaknesses. First, because of the lack of relevant RCTs or prospective studies in this field at present, the relevant conclusions need to be confirmed and expanded by more high‐quality and prospective comparative studies. Second, it is difficult to combine data due to the diversity of the relevant comparative studies, the small number of studies, and missing baseline or outcomes data, this study did not explore the prognosis of the combination of PHA and TACE or the combination of PHA or TACT with other techniques such as radiofrequency, ablation or drugs. Most of these combination regimens were based on PHA or TACE as the primary treatment and used other methods as adjuvants to obtain effect sizes of “> 1”. Our conclusions may therefore also provide potential evidence for differences between these combination regimens. Finally, all of the comparative studies included in this study were from Asia, and we speculate that this may be related to the more common cirrhotic background in Asian patients with HCC. These underlying lesions not only negatively and irreversibly affect the regenerative function of the liver but also contributes to worsening liver function among similar sized volumes, which often impedes hypertrophy of the target lobe and necessitates greater FRLV for second‐stage hepatectomy than in patients with common HCC. Together, these characteristics decrease the surgical effect of PHA [6, 46]. Subgroup analysis of cirrhosis was stopped due to insufficient data; thus, we can only hypothesize that our conclusions may be more applicable to Asian or cirrhotic patients with HCC, whereas for patients from other regions or noncirrhotic groups, PHA may have a better effect than we reported.

## 5 Conclusions

In conclusion, in the face of FRLV‐insufficient HCC patients, PHA is a better option than TACE when extensive radical hepatectomy is no longer possible. This meta‐analysis provides additional evidence that PHA is associated with a good long‐term prognosis and may improve future patient survival and delay tumor recurrence without increasing the rate of adverse events. PHA should be considered in the optimal surgical management of such patients. However, future studies are needed to confirm the present findings regarding the relationship between different types of PHA and TACE.

## Author Contributions

**Wenjie Li:** conceptualization (lead), data curation (lead), investigation (equal), methodology (equal), software (equal), writing – original draft (lead), writing – review and editing (equal). **Hang Li:** data curation (equal), methodology (equal), software (equal), writing – original draft (equal), writing – review and editing (supporting). **Qingyan Kong:** conceptualization (equal), methodology (equal), software (supporting), writing – review and editing (equal). **Fei Teng:** conceptualization (equal), methodology (supporting), writing – original draft (supporting), writing – review and editing (supporting). **Zheyu Chen:** conceptualization (equal), project administration (supporting), resources (lead), supervision (lead), writing – original draft (supporting), writing – review and editing (supporting).

## Disclosure

Data Transparency: All authors ensure that all data and materials as well as software application or custom code support their published claims and comply with field standards.

## Ethics Statement

The authors are accountable for all aspects of the work in ensuring that questions related to the accuracy or integrity of any part of the work are appropriately investigated and resolved. No ethical issues, informed consent, and the care and use of laboratory animals were involved in this study, and institutional ethics committee approval was not needed.

## Conflicts of Interest

The authors declare no conflicts of interest.

## Supporting information


**Table S1.** Operative data and change of liver volume.

**Table S2.** Data of major complications.

**Table S3.** Data of primary outcomes.

## Data Availability

The datasets generated and analyzed during the current study are available in the PubMed, Embase, Cochrane Library, and Web of Science repositories.
