# Supplementary material for: Preoperative Hepatic Augmentation Versus Transarterial Chemoembolization for Hepatocellular Carcinoma With Insufficient Remnant Liver Volume: A Systematic Review and Meta‐Analysis
Source: Cancer Med. 2025 Jul 11;14(13):e71050. doi: 10.1002/cam4.71050 (PMC12246796; doi:10.1002/cam4.71050)
Supplement: Supplementary file 1 — Table S1. Operative data and change of liver volume. [file CAM4-14-e71050-s002.docx]

**Supplementary table 1.** Operative data and Change of liver volume

| Study | Operative time(min):  AP / TA | Blood loss(ml):  AP / TA | Blood transfusion(U):  AP / TA | Interval time(day):  AP / TA | Hospital stays(day):  AP / TA | SLV(ml) | FRLV(ml):  S1p / S2p | FRLV/SLV(%):  S1p / S2p | FRLV increasing(%) | Absolute KGR(ml/day) | Relative KGR(%/day) |
| --- | --- | --- | --- | --- | --- | --- | --- | --- | --- | --- | --- |
| Haoqi et al. | 311.54 ±34.59 / NR | 289.55 ±83.00 / NR | NR / NR | 16(9-24) / NR | NR / NR | 1471.4 (967.1-1980.9) | 362(254-490) /  615(309-781) | 25.3(17.4-29.9) /  42.0(28.9-55.6) | NR | 18.1  (5.8-37.7) | 5.1(2-10.3) |
| Zheng et al. | 244(90-485) /  NR | 350(50–5000) /  NR | 0(0-28) / NR | 12(6-28) / NR | NR / NR | 1203.6 (1029.8-1440.9) | 342(221-488) /  510(384-712) | 26.9(17.9-39.2) /  41.3(33.1-60.5) | 56.8  (8.3-103.5) | 14.4  (5.4-50.6) | 4.9(1.2-14.2) |
| Zhenfeng et al. | 351(213-531) /  NR | 538(100-6000)/  NR | 275(0-2150) / NR | 18(10-54) /  NR | NR / NR | 1013.0 (874.9-1231.6) | 388.8(192.0-477.2) /  578.6(402.1-823.0) | 36.6(19.0-47.4) /  57.6(43.7-85.1) | 64.5  (22.3-221.9) | 22.2  (13.1-42.3) | 2.1(0.8-10.2) |
| JiaHui et al. | 179.5(95-480) /  NR | 350(10–3500) /  NR | 27-18(YES-NO) / NR | 14(8-67) / NR | 11(5-50) /  NR | 1261.7 (1101.2-1578.4) | 301.3(171.9-580.8) /  553.2(455.7-893.6) | 23.6(14.1-36.8) /  43.1(40.0-64.6) | 248.4  (138.9-449.9)  (ml) | 16.9  (2.4-55.5) | NR |
| Lixin et al. | 247.73 ±108.90 / NR | 545.48 ±668.15/ NR | 0.598 ±1.167 / NR | 11.7 ±3.07 /  NR | 14.9±7.3 /  NR | 1241  (1060-1348) | 335.9 ±69.3 /  501.0 ±110.1 | 27.0 ±5.3 /  40.4 ±8.7 | 50.3 ±22.6 | 20.1(2.8-46.6)  20.6 ±12.4 | 5.1(1.1-13.6)  6.0 ±3.6 |
| Chihan et al. | NR / NR | NR / NR | NR / NR | 15.3 ±8.8 /  NR | 29.4±13.1 /  NR | 1219.29 | 448.7 ±169.1 /  658.2 ±170.3 | 36.8 ±10.7 /  54.5 ±10.9 | 52.4 ±26.9 | 17.83 ±10.58 | NR |
| Dong et al. | NR / NR | NR / NR | 2(0–12) / NR | 23.3 ±8.6 /  NR | 20(12-63) /  NR | 1217.89 | 336.5 ±83.7 /  449.4 ±91.9 | 27.6 ±7.2 /  36.9 ±8.1 | NR | NR | NR |
| Gil et al. | NR / NR | NR / NR | NR / NR | 23(20-31) /  46(32-57) | 25(19-42) /  22(19-32) | NR / NR | NR / NR | 34.7 ±7.5 /  46.8 ±6.8 | NR | NR | NR |

Data shown represents mean ± standard deviation or median (minimum-maximum) ; AP, ALPPS & PVE group ; TA, TACE group ; S1p, S1 preoperative ; S2p, S2 preoperative ; NR, not report

SLV, standard liver volume ; FLR, future liver remnant ; KGR, kinetic growth rate
