# Supplementary material for: Preoperative Hepatic Augmentation Versus Transarterial Chemoembolization for Hepatocellular Carcinoma With Insufficient Remnant Liver Volume: A Systematic Review and Meta‐Analysis
Source: Cancer Med. 2025 Jul 11;14(13):e71050. doi: 10.1002/cam4.71050 (PMC12246796; doi:10.1002/cam4.71050)
Supplement: Supplementary file 2 — Table S2. Data of major complications. [file CAM4-14-e71050-s003.docx]

**Supplementary table 2.** Data of major complications

| Study | Clavien-Dindo criteria^†^:  AP / TA | Fever:  AP / TA | Wound problem:  AP / TA | Pleural effusion:  AP / TA | Ascites:  AP / TA | Gastrointestinal symptoms:  AP / TA | Postoperative bleeding:  AP / TA | Liver lesions:  AP / TA | Bile leak:  AP / TA | PHLF ^‡^:  AP / TA | Postoperative  complications：  AP / TA | Severe  Complications*： AP / TA |
| --- | --- | --- | --- | --- | --- | --- | --- | --- | --- | --- | --- | --- |
| Haoqi et al. | 10-2(≤Ⅲa-≥Ⅲb) /  12-0(≤Ⅲa-≥Ⅲb) | NR /  4(18.18%) | 2(9.09%) /  NR | 5(22.73%) /  NR | NR / NR | 1(4.55%) /  6(27.27%) | NR / NR | NR / 1(4.55%) | 2(9.09%) /  NR | NR / NR | 12(54.55%) /  12(54.55%) | 2(9.09%) /  0(0.00%) |
| Zheng et al. | 8-15-6-0-3-3  (I-II-IIIa-IIIb-IV-V) /  NR | NR / NR | NR / NR | NR / NR | NR / NR | NR / NR | NR / NR | NR / NR | NR / NR | 16-5-3 /  NR | 23(56.10%) /  NR | 9(14.63%) /  NR |
| Zhenfeng et al. | 7-8-3-2(I-II-III-IV) /  NR | NR / NR | NR / NR | NR / NR | NR / NR | NR / NR | NR / NR | NR / NR | 1(5.00%) /  NR | 3-16-1 /  NR | 20(100.00%) /  NR | 5(25.00%) /  NR |
| JiaHui et al. | 9-1-1-1  (≤IIIa-IIIb-IV-V) /  NR | 1(2.22%) /  26.60% | NR / NR | 1(2.22%) /  1.60% | 4(8.89%) /  5.40% | 2(4.44%) /  52.2% | 1(2.22%) /  NR | NR /0.5% | 1(2.22%) /  NR | 2(4.44%) /  5.40% | 12(26.67%) /  91.70% | 3(6.67%) /  24(13.04%) |
| Lixin et al. | 0-2-1-0-0  (I-II-III-IV-V) /  NR | NR / NR | NR / NR | 1(5.00%) /  NR | NR / NR | NR / NR | NR / NR | NR / NR | NR / NR | 2(10.00%) /  NR | 3(15.00%) /  NR | 1(5.00%) /  NR |
| Chihan et al. | 3-6-1-1-0-2  (I-II-IIIa-IIIb-IV-V) /  3-1-0-0-0-0  (I-II-IIIa-IIIb-IV-V) | NR / NR | NR / NR | NR / NR | NR / NR | NR / NR | NR / NR | NR / NR | NR / NR | NR / NR | 13(65.00%) /  4(20.00%) | 3(15.00%) /  0(0.00%) |
| Dong et al. | NR / NR | NR / NR | NR / NR | 2(6.25%) /  NR | NR / NR | NR / NR | NR / NR | 2(6.25%) /  NR | 1(3.13) /  NR | 1(3.13%) /  NR | 6(18.75%) /  NR | NR / NR |
| Gil et al. | 7-3-2-1-3  (≤II-IIIa-IIIb-IV-V) /  4-2-1-0-0  (≤II-IIIa-IIIb-IV-V) | NR / NR | 3(7.89%) /  NR | 0(0.00%) /  2(7.14%) | 4(10.53%) /  1(3.57%) | NR / NR | 4(10.53%) /  1(3.57%) | NR / NR | 3(7.89%) /  1(3.57%) | 1(2.63%) /  NR | 16(42.11%) /  7(25.00%) | 9(23.68%) /  3(10.71%) |

Data shown represents mean ± standard deviation or median (minimum-maximum) ; AP, ALPPS & PVE group ; TA, TACE group ; NR, not report

^†^ Postoperative complication is graded according to the Clavien-Dindo classification of surgical complications, Clavien-Dindo criteria=(Grade I-II-IIIa-IIIb-IV-V)

^‡^ PHLF, post-hepatectomy liver failure, Liver failure is graded according to the International Study Group of Liver Surgery(ISGLS) classification, PHLF=(Grade A-B-C)

*Severe complications were defined as Clavien-Dindo grade III / IIIb or higher
