# Supplementary material for: Preoperative Hepatic Augmentation Versus Transarterial Chemoembolization for Hepatocellular Carcinoma With Insufficient Remnant Liver Volume: A Systematic Review and Meta‐Analysis
Source: Cancer Med. 2025 Jul 11;14(13):e71050. doi: 10.1002/cam4.71050 (PMC12246796; doi:10.1002/cam4.71050)
Supplement: Supplementary file 3 — Table S3. Data of primary outcomes. [file CAM4-14-e71050-s001.docx]

**Supplementary table 3.** Data of primary outcomes

| Study | 1. day Mortality:   AP / TA | Median OS(month):  AP / TA | 1. year OS(%):   AP / TA | 3-year OS(%):  AP / TA | 1. year OS(%):   AP / TA | 1. year DFS(%):   AP / TA | 3-year DFS(%):  AP / TA | 5-year DFS(%):  AP / TA |
| --- | --- | --- | --- | --- | --- | --- | --- | --- |
| Haoqi et al. | 1(4.55%) /  NR | 19.9 / 23.9 | 71.4 / 76.2 | 11.6 / 38.1 | 4.8 / 4.8 | 57.1 / NR | NR / NR | 0.0 / NR |
| Zheng et al. | 5(11.11%) /  NR | None / 12.0 | 64.2 / 50.0 | 60.2 / 7.1 | NR / NR | 47.6 / 5.0 | 43.9 / 0.0 | NR / NR |
| Zhenfeng et al. | 1(5.00%) /  NR | None / 8.5 | 70.0 / 40.0 | 57.4 / 15.6 | NR / NR | 60.0 / 35.0 | 43.0 / 6.0 | NR / NR |
| JiaHui et al. | 4(7.41%) /  NR | 18.7 / 11.2 | 60.5 / 46.6 | 31.9 / 16.5 | 26.3 / 0.0 | 50.5 / NR | 22.4 / NR | 19.2 / NR |
| Lixin et al. | 3(13.04%) /  NR | 8.0 / 30.0 | 50.0 / 84.6 | 20.1 / 44.0 | 10.0 / NR | 27.8 / NR | NR / NR | 0.0 / NR |
| Chihan et al. | 2(10.00%) /  0(0.00%) | 27.4±3.8 / 13.5±1.2 | 69.6 / 55.0 | 58.9 / 5.0 | NR / NR | NR / NR | NR / NR | NR / NR |
| Dong et al. | 0(0.00%) /  0(0.00%) | None / 55.5 | 96.9 / 90.6 | 71.9 / 59.7 | 71.9 / 45.6 | 59.4 / 53.7 | 40.6 / 31.0 | 37.2 / 24.3 |
| Gil et al. | 4(10.53%) /  1(3.57%) | 7.6 / 4.8 | 76.5 / 78.7 | 68.6 / 54.8 | 57.7 / 45.1 | 71.6 / 58.5 | 49.2 / 40.0 | 49.2 / 40.0 |

Data shown represents mean ± standard deviation or median (minimum-maximum) ; AP, ALPPS & PVE group ; TA, TACE group ; NR, not report

OS, overall survival ; DFS, disease-free survival
